# A hybrid group-based movie recommendation framework with overlapping memberships

**DOI:** 10.1371/journal.pone.0266103

**Published:** 2022-03-31

**Authors:** Yasher Ali, Osman Khalid, Imran Ali Khan, Syed Sajid Hussain, Faisal Rehman, Sajid Siraj, Raheel Nawaz

**Affiliations:** 1 Department of Computer Science, COMSATS University Islamabad, Abbottabad Campus, Pakistan; 2 Leeds University Business School, University of Leeds, Leeds, United Kingdom; 3 Manchester Metropolitan University, Manchester, United Kingdom; Indian Institute of Technology Patna, INDIA

## Abstract

Recommender Systems (RS) are widely used to help people or group of people in finding their required information amid the issue of ever-growing information overload. The existing group recommender approaches consider users to be part of a single group only, but in real life a user may be associated with multiple groups having conflicting preferences. For instance, a person may have different preferences in watching movies with friends than with family. In this paper, we address this problem by proposing a Hybrid Two-phase Group Recommender Framework (HTGF) that takes into consideration the possibility of users having simultaneous membership of multiple groups. Unlike the existing group recommender systems that use traditional methods like K-Means, Pearson correlation, and cosine similarity to form groups, we use Fuzzy C-means clustering which assigns a degree of membership to each user for each group, and then Pearson similarity is used to form groups. We demonstrate the usefulness of our proposed framework using a movies data set. The experiments were conducted on MovieLens 1M dataset where we used Neural Collaborative Filtering to recommend Top-k movies to each group. The results demonstrate that our proposed framework outperforms the traditional approaches when compared in terms of group satisfaction parameters, as well as the conventional metrics of precision, recall, and F-measure.

## Introduction

### Background

The last two decades have witnessed a growth in data due to increased use of online applications including e-commerce, online social networks, and multimedia streaming. The information on websites is overwhelming due to which users often find it difficult to access the content of their choice. Information overload is an increasing problem of knowledge engineering that cannot be ignored as users are more interested in finding only relevant information.

Recommender Systems (RS) [1, 2] are mathematical models developed in late 90s to compute recommendations for a user that are closely related to the user’s preferences. After the announcement of Netflix Prize, RS have received great attention in industries and academia [3]. Numerous factors are involved while computing recommendation for a user, such as a user’s interests, mood, tastes, and similarity with other users, to name a few [4]. The existing literature takes into account the aforementioned factors to improve the recommendation quality. Generally, the existing schemes can be categorized as Collaborative Filtering (CF), Content Based Filtering, and Hybrid Models [5]. The CF based methods consider like-minded users and then recommend items by aggregating the preferences of similar users, while content-based models perform recommendations based on similarity of items that the user has interacted with in the past [6]. Hybrid recommender systems combine the recommendations of various approaches, and then recommend Top-k items.

### Research problem

Most RS were designed to provide recommendations for individual users. However, people are more social, and activities in group become an important part of daily life [7]. For instance, people find it more entertaining to visit restaurants, picnic spots, trip sites, or watch movies in groups [8, 9]. As more and more people are getting connected on online social networks, such as Facebook, Instagram, Twitter, etc. new avenues of research are opened in the domain of group recommender systems. In recent years, the availability of numerous movie streaming companies, such as Netflix, Amazon Prime, HBO Max, Disney+, and so on, have attracted the interest of research community towards movie recommender systems. Most of the existing recommender systems focus on recommending movie to individual users based on their individualized preferences and past ratings. However, recommending movie to a group is still a challenging problem, as it is constrained by the numerous factors, such as conflicting preferences, timing, and moods of individual members in the group [7]. Generally, users’ preferences are contextually dynamic in nature. For instance, the point of interest (POI) preferred for friends may be different from the POI preferred for families. Moreover, users may have similar preferences for one locality and diverse preferences for other locality.

### Motivation

Several existing works consider locality information while computing recommendations. For example, Ramesh *et al*. [10], proposed a Hierarchical Contextual Location Recommendation System termed as HiRecS. They proposed hierarchical aggregation technique, where the root node represents Top-k recommended locations. The subsequent levels split the preferences based on different localities. The authors applied hierarchical clustering to cover the dynamic preferences of users. However, HiRecs is computationally expensive in terms of processing. In [11], the authors proposed an influence based group recommender framework. The authors created a trust metric and identified leader in a group to calculate influential ratings of group members on items and applied average aggregation to recommend Top-k items. Moreover, the authors used memory based technique and calculated influential ratings in order to recommend movies. However, the memory based approaches are negatively affected with data sparsity.

In recent years, several deep learning based models are proposed that help in better capturing of hidden features and relationships between user and items [12, 13]. The authors in [14] proposed a deep learning algorithm to recommend movies to a group of users. They considered the user ratings, user consumption ratio, and user preferences while building the system. K-means clustering is applied on users ratings to group users with similar preferences in movies. However, k-means clustering forms spherical clusters and does not deal with arbitrary shaped data. Dutta *et al*. proposed a model for recommending movies to group of users by extracting the semantic information, such as tags assigned to each movie by users [15]. The proposed model relied on semantic information, i.e., it did not cope with noisy tags. In [16], the authors proposed a Top-N-Rec model that uses the content-based and collaborative filtering to generate parallel recommendations, and then recommendation from both approaches are merged to generate the final recommendations. However, the proposed model does not perform well with sparse data. Several works have been proposed in recent years to utilize transformer based methods that perform Natural Language Processing (NLP) tasks on users’ feedbacks to compute ratings (e.g., [17–19]). However, the applications of transformer methods on group-based movie recommender systems have not been explored much.

It can be observed from the above discussion that despite significant progress, the traditional group recommender systems suffer from performance issues, such as data sparsity, scalability, and cold-start problem [20] as they mostly target a single type of relation. For instance, Boltzmann machine considers either user-to-user or user-to-item relation. Alternatively, matrix factorization explicitly captures interactions among user to items [21]. However, with sparse data this results low quality predictions. A key factor is the selection of similarity metrics to form groups. Most of the existing group recommender systems utilize traditional methods, such as cosine similarity, K-Means, Jaccard similarity, etc. for creating groups (e.g., [15, 22, 23]). Such approaches result in less efficient group formation when the dataset is sparse [11].

In recent years, there has been a growing inclination towards model-based group recommender systems. Due to the implicit feature learning of neural networks, researchers have applied these models for solving recommendation problems [24]. Most of the existing model-based schemes utilize matrix-factorization methods whose estimation based mechanisms result in low prediction accuracy in cold start and sparsity scenarios [25–27]. In [28], the authors captured the fairness among group members by using SVD++ model. However, the model lacked in capturing the implicit hidden features between users that negatively affected the prediction accuracy.

The existing literature contains limited work on group-based movie recommender systems. Moreover, most of the current studies consider a user to be part of a single group only, but in reality, a user may be associated with multiple groups. For instance, a user may have different preferences in watching a movie with friends or family. The existing group recommender system employ various methods, such as k-clique, cosine similarity, Jaccard similarity, etc., to form groups. Such methods allow a user to be part of distinct groups, whereas in real-world scenarios, a user may be part of overlapping groups. These scenarios are handled in our proposed movie-based group recommender system.

### Contributions

**Fig 1:**
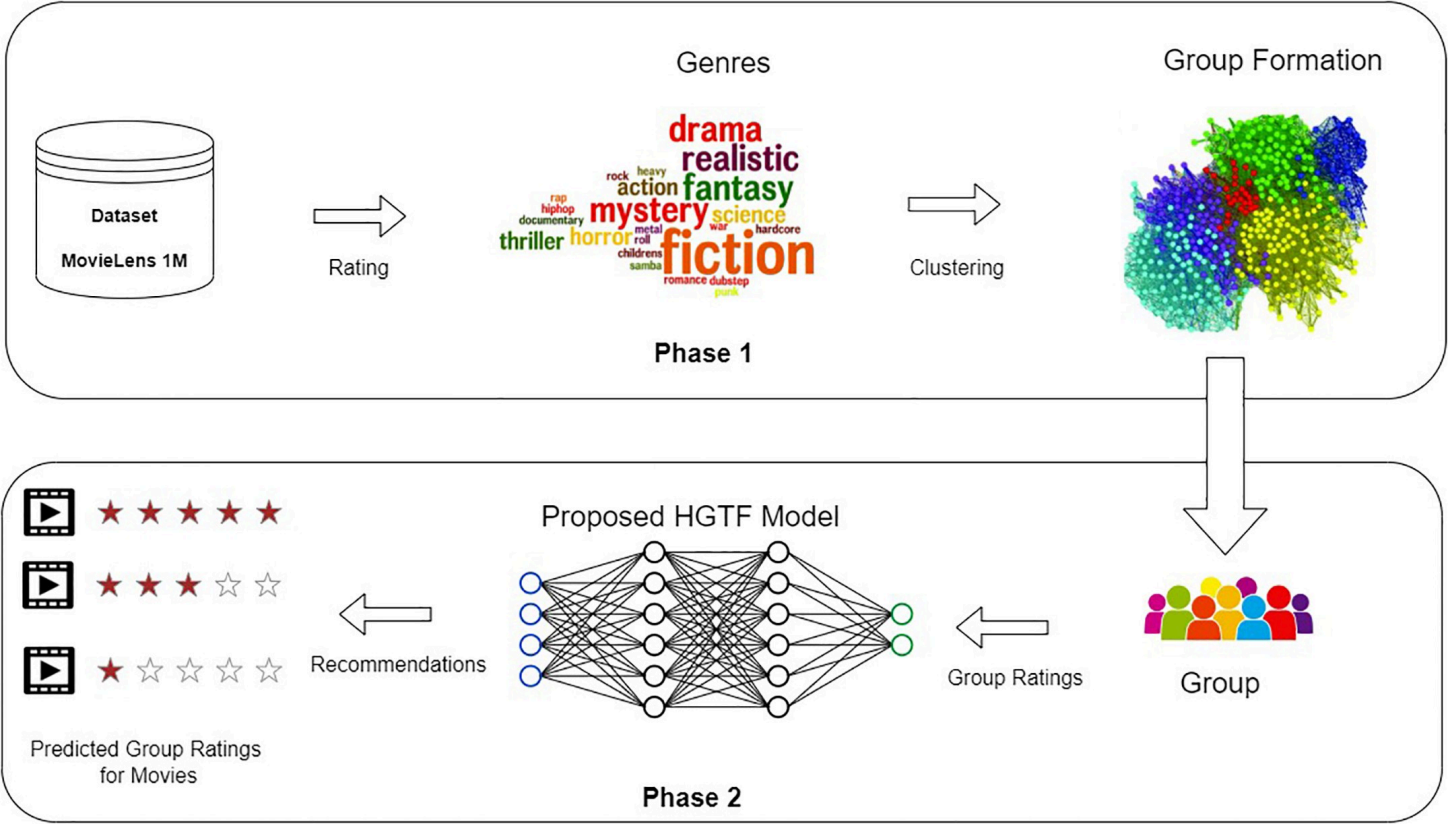
Group recommender system.

To overcome the aforementioned issues, we propose a novel Hybrid Two-phase Group recommender Framework (HTGF) for movie recommendations. The proposed framework makes use of deep neural networks to efficiently perform model learning based on explicit preferences of members as their ratings, movies’ features, and implicit preferences, such as interaction of group members. The intra-group similarity presents a unique challenge in group recommendations as the users’ interest may overlap onto different groups. To improve intra-group similarity in group formation, we apply a combination of Fuzzy c-means clustering (FCM) and Pearson Correlation Coefficient (PCC) [11], which helps in diverse membership degree of each and every individual, concerned with distinct clusters. The model exhibits some degree of generalization by allowing a user to be part of multiple groups, which has not been the case in the existing movie-based group recommender systems. The data sparseness issue is addressed in the proposed model by utilizing latent factors of users and movies to overcome negative effects of sparsity, thereby improving the prediction accuracy. Fig 1 describes the general overview of group recommendation process consisting of two phases.

The Phase 1 is group formation that uses a combination of PCC and FCM clustering thereby allowing the users to have multiple groups. The Phase 2 is group recommendation in which Neural Collaborative Filtering (NCF)-based approach is used to predict the ratings of unrated items of group [29], and average aggregation strategy is applied to recommend Top-k items. The main contributions of our work are summarized as follows.

- We utilize deep learning to develop a group-based movie recommender system, HTGF, which efficiently captures the implicit and explicit features of movie and preferences of end users.
- We address the intra-group similarity issue by using a combination of FCM and PCC, and the proposed system allows a user to be part of different groups.
- To efficiently capture the implicit preferences between group members and to improve prediction accuracy, we utilized NCF.
- To address data sparsity, we use the latent vectors of users and movies by converting them into low dimensional vector space, which are input to NCF to improve the prediction accuracy.
- An enhanced average aggregation strategy is presented to generate top-K recommendations.
- We present statistical tests to display the statistical significance of our results.
- We performed comparisons with existing schemes and results indicate that our model outperforms the baseline approaches.

The rest of the paper is structured as follows. Section II presents the related work. Section III presents the overview of HTGF, and Section IV presents the proposed model. In Section V, we present the performance metrics with the experimental results, and Section VI concludes the paper.

## Related work

In this section, some the recent works on group recommender systems and their shortcomings are discussed. Initially, we discuss the approaches used for group formation. Then, some of existing proposals for group recommendation are presented, and finally transformer-based approaches using NLP for recommendations are discussed. A comparison table is included at the end of the section presenting a concise summary of state-of-the-art and their limitations.

### Group formation

Most of the existing datasets, such as MovieLens [30], Yelp [31], Trip Advisor [32], and CAMRa2011 [33] do not contain any explicit information about members’ relation with each other. More specifically, there is no predefined or ready-made group membership information available in such datasets. Researchers mostly employed various clustering techniques to form groups. The clustering methods usually applied in group recommender systems consists of partition-based methods [34, 35], such as k-mean and C-boost [36, 37], hierarchy based methods [38, 39], e.g., bottom up clustering [40], Density based methods like DBSCAN, Grid based [41], and model based [42]. Despite having benefits, the aforementioned models have some limitations.

For example, in k-means, advanced selection of cluster centers makes it simple and efficient, but only spherical clusters can be formed. In the bottom-up clustering [38, 39], each data point is known as a cluster, then distance between all the data points is measured and combined until all the clusters are merged into one cluster. However, the efficiency of bottom-up clustering is low because the time complexity for clustering is very high. DBSCAN bests suits for arbitrary shaped clustering, but it has high cost of time. Moreover, the aforementioned clustering approaches for group formation associate a user with a single group but in reality a user may have multiple groups. To address these limitations, our proposed model assigns a user to different groups, so that a users’ preferences are properly reflected in a group based on his/her membership score.

### Group recommendation

Recommender systems have been widely applied in various domains such as, medical diagnosis, e-tourism, and multimedia streaming applications, etc. In literature, most of the studies were conducted on individual recommendations, but limited research is performed on group recommendation [43]. At present, group recommendations can be generally classified into Memory-based and Model-based [44, 45]. Memory-based methods in group recommendations are further divided into Preference Aggregation (PA) method [46] and Score-Aggregation (SA) method [47]. The PA method aggregates the profiles of all group members into a single group profile, and then generate recommendation for the group. The SA method aggregates the scores based on a predefined strategy to predict group preferences. Common aggregation strategies are average (AVG) [48], most pleasure (MP) [49], least misery (LM) [50], etc. However, aggregation methods have some shortcomings: (a) they cannot capture the implicit preferences among group members and (b) it is hard to construct group preference model effectively through aggregation strategies due to sparsity of user explicit feedback.

In past few years, a few model-based methods are proposed to capture the implicit preferences among group members. Minjae *et al*. [23] proposed a deep learning algorithm based on Recurrent Neural Network (RNN), which learns the movie consumption patterns of users, and then recommend movies according to extracted features. They created groups by measuring the similarity between group members based on ratings of similar movie preferences [23]. After performing clustering, the authors applied RNN to learn the movie consumption behavior of each specific group of users. By considering the shift in tastes over time, the authors enhanced prediction accuracy. However, the proposed model predicted a limited set of movies with less accuracy [23]. The authors in [25] evaluated aggregation strategies: average and most pleasure, on two baseline models Alternating Least Squares (ALS) and Singular Value Decomposition (SVD). For clustering, they applied cosine similarity, and for recommendation they used two baseline models. Based on results they concluded that average strategy produces better results than MP, and SVD model predicted more accurate ratings than ALS.

In recent years, the research community has widely applied deep learning to the recommender systems, which helps in capturing hidden features and implicit relationships between users and items. Huang *et al*. proposed a mutli attention-based group recommender model that considered preference interactions and sociality between group members [51]. The proposed system utilized multiattention-based neural network model to train group feature and preference learning modules for groups on items. The deep semantic feature for each group is learned. However, the system is complex as it utilizes a neural network for each social attribute of the members. Moreover, the recommendation performance is degraded by using lesser number of sub-features.

It can observed from the aforementioned discussion that the deep learning based models are mostly applied to recommender systems to provide individual recommendations. However, the applications of deep learning to group recommender systems are under-explored due to the specific challenges of group recommender systems, where preference of each member has to be taken care of to estimate an overall recommendation for the group. Most of the existing deep learning based approaches have some performance deficiencies caused by their failure to capture implicit interactions among group members and poor preference estimations between groups and their members. To address these issues, the proposed group recommendation framework HTGF uses NCF that considers latent features to capture implicit relationships among group members and movies. This mechanism improves the prediction accuracy and minimizes the Root Mean Square Error (RMSE) and Mean Absolute Error (MAE).

### Transformer-based approaches

Over recent years, the increase in social media and e-commerce websites have initiated a paradigm shift in recommender system research towards transformer-based approaches that utilize NLP tasks to extract users’ preferential information [52–56]. For instance, Aipe *et al*. proposed a sentiment-aware recommendation model to develop a patient assisted health-care system [17]. The proposed model performs sentiment-based scoring on the information extracted from the medical forum. A deep learning model comprising of CNN is proposed for sentiment analysis, followed by LSTM for the classification of data into specific sentiment class. Top-n similar posts are retrieved for a blog classified with positive sentiment, and a probabilistic model is developed to suggest treatments for specific health condition. However, the model can suffer with anomalies due to lack of any standard procedure for dataset annotation. Moreover, no details are provided about the source of the dataset and the quantitative evaluation of the suggested modules.

In [18], the authors proposed a financial product recommendation system, namely R-Transformer, based on transformer approach. The proposed system generates user and financial product state vectors based on historical interaction sequence of users and financial products. The resultant vectors are high-dimensional and sparse, and therefore a pre-processing phase is introduced to reduce the dimensionality using autoencoder. The processed data is input to the transformer layer to compute user financial products’ score vector by utilizing time-series information. However, the source of financial data and its attributes are not clearly defined. The authors in [19] performed a comparison of content-based recommendation systems that are based on: (a) Vector Space Model (VSM), (b) Bidirectional Recurrent Neural Networks (BRNN), and (c) a semantic-aware recommendation system that uses Linked Open Data (LOD)-based textual descriptions of items, and Bidirectional Encoder Representations from Transformers (BERT) for language modeling. The BERT textual classification is performed using the paragraphs as input, and cold start problem is addressed by increasing the availability of textual data. The results indicated better performance of BERT-based content recommendation system on movie data.

Lin *et al*. compared the performance of popular open-source machine learning libraries, such as Scikit-learn and TensorFlow [57]. The authors evaluated the advantages, error measures, and processing times of the aforementioned tools. It was concluded in the study that Scikit-learn could be a better choice for traditional machine learning approaches, and TensorFlow is good for neural networks. In [58], the authors proposed a method of automatically computing features from a video file by using MPEG-7 visual descriptors and deep learning-based hidden layers. The aim is to analyze a movie stream content and extract a set of low-level features, which can be used to make personalized recommendations as per a user’s preferences. However, the process requires high computation and processing time to extract features from full-length movies, making it a computationally expensive task. Fu *et al*. proposed a CF-recommendation model based on deep learning [21]. The model consists of first building a user-item low dimensional vector by using word embedding in NLP based on context of the user. The context is captured from user-user co-occurrence information in the past. Similarly, the knowledge of items is obtained by observing the past item-item co-occurrence. In the second phase, feed-forward neural network is developed to generate prediction from pre-learned embedded vectors of users and items. The model attempted to improve the prediction accuracy at the higher cost of computational complexity.

The aforementioned recommendation systems are designed with an aim to perform individual recommendations, whereas our main focus is towards group recommendation. Moreover, the models employed sentiment analysis to compute ratings which can lead to increased complexity in the case of group recommendation systems, where certain tradeoffs need to be taken into account to reach a consensus among group members. Table 1 presents a summary and limitations of recent state-of-the-art schemes.

**Table 1:** Comparisons of state of the art schemes.

Year	Ref.	Objective	Similarity Measure	Model	Limitations
2021	[59]	Dimensionality reduction technique is utilized to classify users of same interest	Euclidean distance and PCA	K-mean	Interaction among group members is not addressed; uses memory-based technique to recommend movies
2021	[60]	Recommended movies by using a combination of K-mean and FF	Mini-batch K-mean	Field-aware factorization machine (FFM)	Mini batch K-mean does not deal with arbitrary shaped data
2020	[61]	Users’ personal information is utilized to solve cold-start and data sparsity problem	k-Clique, Cosine Similarity	Ranking measure method	Uses resource intensive memory-based technique to recommend movies
2020	[25]	Evaluation of group recommendations strategies	Cosine Similarity	ALS and SVD	Models does not capture the implicit preferences of group members
2019	[23]	Model incorporates the change of preferences over time	Pearson correlation coefficient	Recurrent Neural Network	Low prediction accuracy and distinct users in groups
2019	[15]	Used semantic information present in tags to make recommendations	Random Groups	WordNet	Noisy Tags
2019	[22]	Flexible size user preferences in group recommendations	Jaccard Similarity coefficient	Aggregation Strategies (Least misery and Average)	Only consider users explicit feedback
2018	[14]	Consider user consumption ratio in group recommendations	K-mean	Pattern Recognition Network	Only spherical clusters can be formed
2017	[62]	Effect of order in group recommendations	Jaccard Similarity coefficient	Aggregation Strategies (Least misery, Average)	Only consider users explicit feedback

## HTGF framework

**Fig 2:**
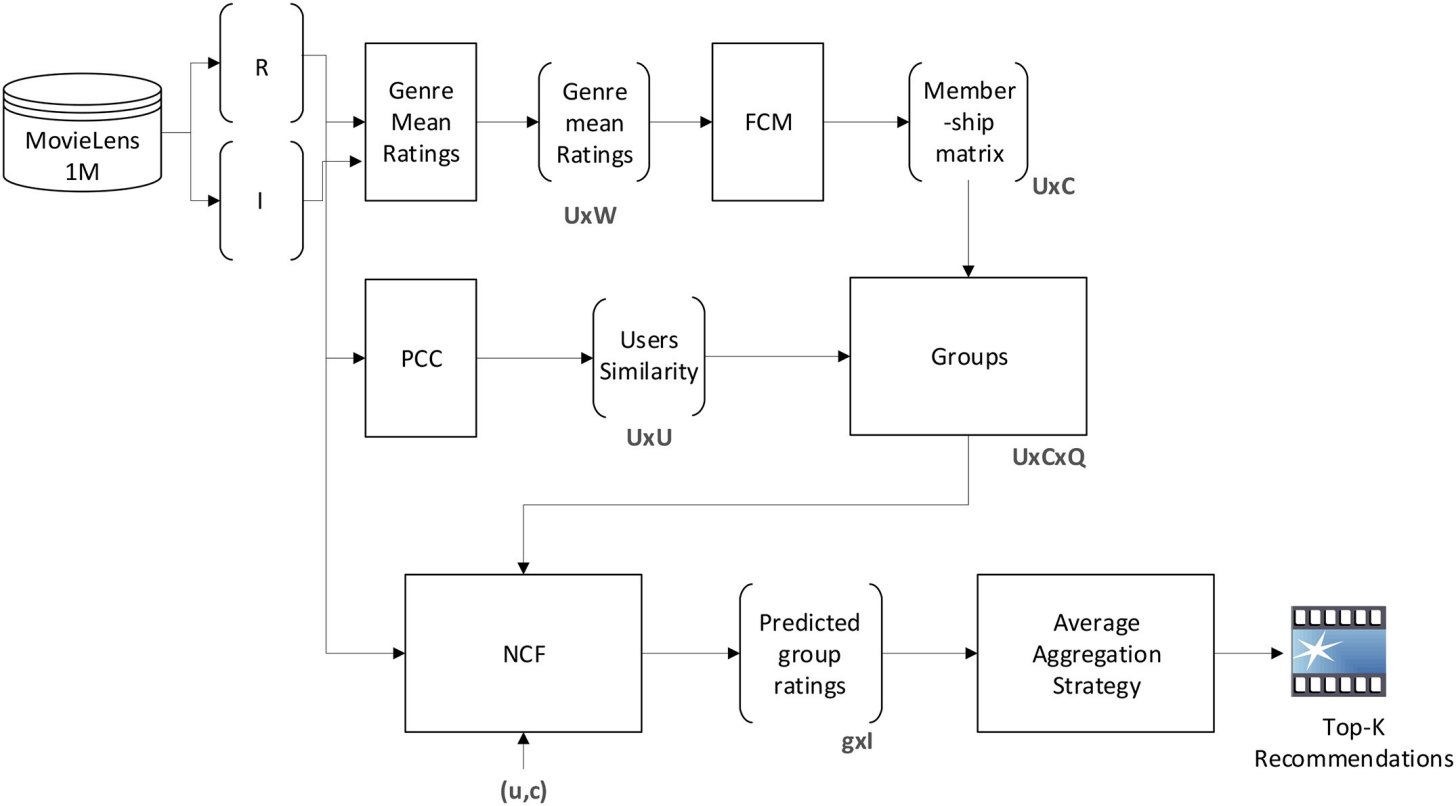
System overview.

The overall architecture of our proposed HTGF is shown in Fig 2. The whole process consists of two phases: (a) group formation and (b) group recommendation. Group recommendation is a complex process and many factors can effect the performance of group recommendations. Selecting an appropriate similarity measure to form group is the key component of any group recommendation system.

In group formation, a user may be associated with multiple groups. Users’ preferences vary from group to group. For instance, a user has different preferences while watching a movie with friends, and may have different interests while watching movie with family. We used FCM [63] to represent user to group associations. FCM assigns a membership value to each user corresponding to each group. Initially, we have calculated the mean genre ratings of users, and then apply FCM to cluster users into multiple groups by assigning membership value to users in each cluster. After calculating the membership value, we applied PCC to select the highly similar users to a user *u*. Top-k users similar to *u* were selected to form group. Group rating matrix is sparse, so to predict the ratings of unrated movies, we apply NCF [29] to group ratings and train it on group members ratings. After learning the implicit preferences of group members, NCF predicts the ratings of movies. To compute the group rating on item *i* we apply average aggregation strategy, as shown in Fig 2. The average ratings of every movie is calculated, and based on that Top-k movies are recommended to the group. Table 2 shows the notations and their meanings used in the subsequent text.

**Table 2:** Notations and their meanings.

Notation	Meaning
*U*	Set of users
*I*	Set of items
*R*	Rating matrix
*W*	Set of genres
*C*	Set of clusters
*Q*	Set of groups
*X*	Mean genre ratings
*M*	Membership matrix
*r*_*ui*_	Rating of user *u* on item *i*
*r*_*u*_	Ratings of user *u*
*g*	Group of users

## Proposed model

The existing datasets do not have any explicitly embedded information to represent groups. In literature, researchers proposed various clustering methods to form groups [64]. One of the popular and well-known algorithm is FCM, as it generates better results than k-means [11]. The FCM is applied to cluster users into groups i.e., friends or family. For instance, a person has different preferences while watching a movie with friends, and may have a different taste while watching a movie with family. In FCM, users are split into *c* number of clusters by allowing a user to have membership corresponding to each cluster. The objective function *O* of FCM is as follows.

$$\begin{array}{c} O=\sum_{i=1}^{c}\sum_{k=1}^{n}\mu_{ik}^{m}d_{ik}^{2}. \end{array}$$ (1)

$$\begin{array}{c} d_{ik}^{2}={|x_{k}-v_{i}|}^{2}. \end{array}$$ (2)

$$\begin{array}{c} \sum_{i=1}^{c}\mu_{ik}=1,\forall k=1,\ldots ,n. \end{array}$$ (3)

Where *c* is the total number of clusters, *n* is the total number of users, *m* is the fuzziness parameter (1.25 ≤ *m* ≤ 2). Eq (3) states that the total membership value of each user corresponding to each group is one. The objective function must be minimized.

$$\begin{array}{c} v_{i}=\sum_{k=1}^{n}\mu_{ik}^{m}x_{k}. \end{array}$$ (4)

$$\begin{array}{c} \mu_{ik}={\left[\sum_{i=1}^{c}{\left(\frac{d_{ik}}{d_{jk}}\right)}^{\frac{2}{m-1}}\right]}^{-1}. \end{array}$$ (5)

Where *v*_*i*_ is the *i*^*th*^ cluster center, and *μ*_*ik*_ ∈ *M*, is the membership value of user *k* to the cluster *i*. A detailed explanation of Fuzzy C-means can be found in [63].

After forming clusters, the Pearson similarity is computed between users to improve intra-group similarity. We used Pearson Correlation Coefficient (PCC) because it is one the well-known method used for similarity measurement. In order to measure the similarity between two users *u* and *v*, PCC uses the common users ratings on item to calculate similarity [65]. Pearson Similarity can be defined as follows.

$$\begin{array}{c} Sim_{u,v}=\frac{\left(\sum_{i\in I}(r_{u,i}-\bar{r_{u}})(r_{v,i}-\bar{r_{v}})\right)}{\left(\sqrt{\sum_{i\in I}{\left(r_{u,i}-\bar{r_{u}}\right)}^{2}}\sqrt{\sum_{i\in I}{\left(r_{v,i}-\bar{r_{v}}\right)}^{2}}\right)}. \end{array}$$ (6)

Pearson similarity ranges from [−1, +1]. Negative correlation indicates that users are not similar, and positive correlation indicates that users are highly similar. The similarity computation between user *u* and *v* in cluster *k* is defined as:

$$\begin{array}{c} S_{uvc}=Sim_{u,v}\times \mu_{vc}. \end{array}$$ (7)

**Fig 3:**
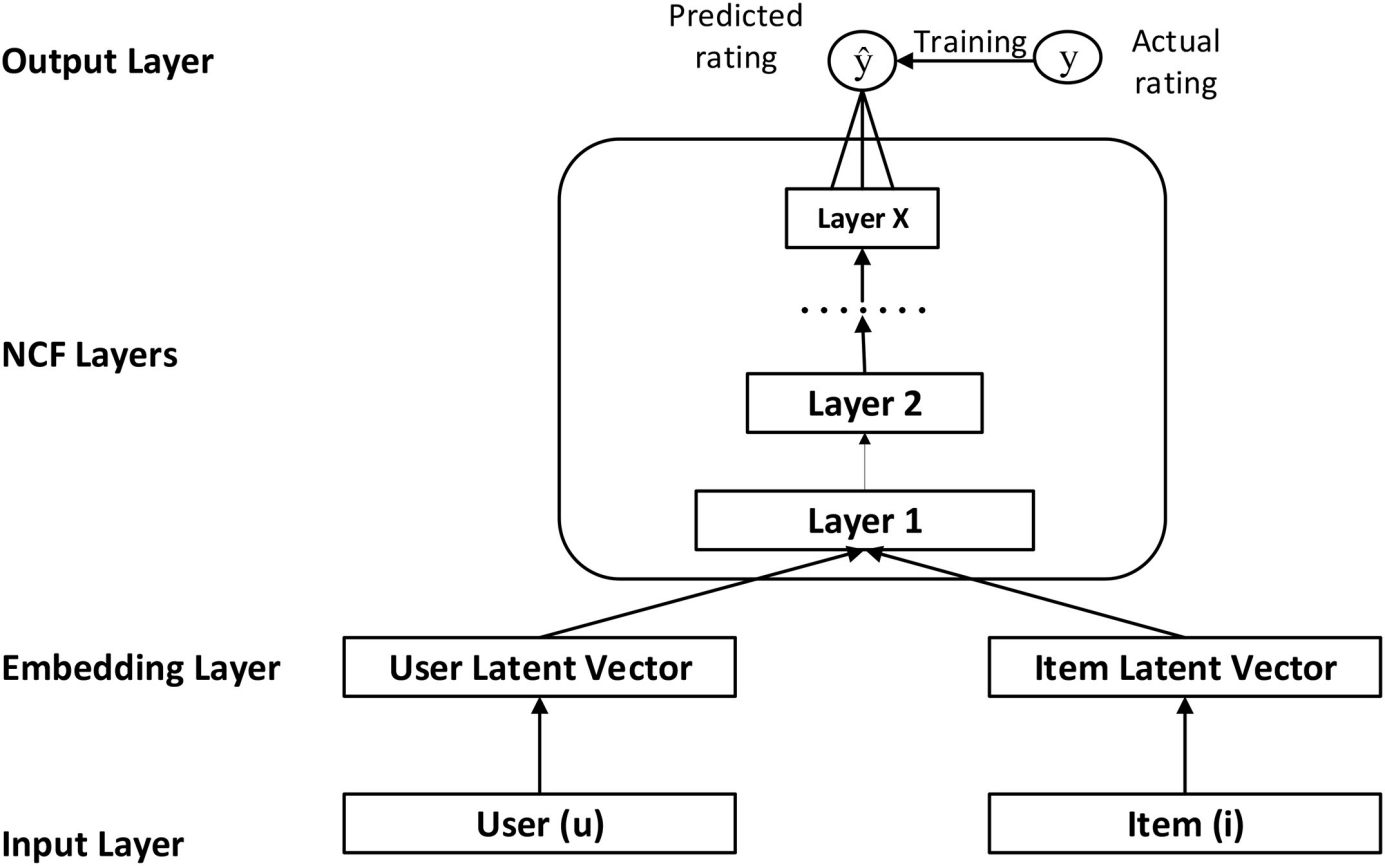
Neural Collaborative Filtering.

Where *S*_*uvc*_ is the product of similarity between users *u* and *v*, and the membership of *v* in cluster *c*. Based on Eq (7), the Top-k similar users are selected to form a group. After forming group, NCF [29] is used to predict the unrated movies of group members. Fig 3 describes the NCF framework. NCF is a layered model as it consists of an input layer, hidden layers, and output layer.

In output layer, sigmoid activation function is used. The input layer consists of two feature vectors $v_{u}^{U}$ and $v_{i}^{I}$ that describes the user *u* and movie *i*. The parameter ${\hat{y}}_{ui}$ is the predicted rating of user *u* on movie *i* and can be defined as follows [29].

$$\begin{array}{c} {\hat{y}}_{ui}=f(P^{T}v_{u}^{U},Q^{T}v_{i}^{I}|P,Q,\Theta_{f}) \end{array}$$ (8)

Where *P* ∈ *R*^*U*×*K*^ and *Q* ∈ *R*^*I*×*K*^ are the latent factors of users and movies. Θ_*f*_ denotes model parameters, *f* is layered neural network. The loss function of NCF is defined as follows.

$$\begin{array}{cc} L & =-\sum_{(u,i)\in \tilde{y}}log{\hat{y}}_{ui}-\sum_{(u,j)\in ý}log(1-{\hat{y}}_{j})\\ & =-\sum_{(u,i)\in ý\cup \tilde{y}}y_{ui}log{\hat{y}}_{ui}+(1-y_{ui})\;log(1-{\hat{y}}_{ui}). \end{array}$$ (9)

Where $\tilde{y}$ is the observed interaction, and *ý* is the unobserved interaction between the user and movie. Eq (9) is the objective function of NCF. In order to minimize objective function, Stochastic Gradient Descent (SGD) is used. After predicting the unrated movies ratings, we apply average aggregation strategy on group ratings and recommend Top-k movies to the group. The following equation is used to calculate average group rating:

$$\begin{array}{c} G_{r_{i}}=\frac{\sum_{a=1}^{j}r_{a,i}}{j}. \end{array}$$ (10)

Where *j* be the number of users in a group, *r*_*ai*_ is the rating of user *a* on movie *i*, and $G_{r_{i}}$ is the group rating on movie *i*.

The pseudocode for *Group Formation* is presented in Algorithm 1.

1. *Initializations (Line 1−Line 4)*: The algorithm takes as input the following parameters: (*a*) ratings of users, (*b*) Items; consisting of movies’ title and genres, (*c*) k-users, which is the number of users in a group. In Line 1, genres are extracted from Movies. Line 2 − 3 initialize the mean genre matrix and initial membership matrix to 0. Line 4 calculates the initial clusters’ centers as defined in Eq (4).
2. Average genre ratings are calculated in Line 5−Line 14, where *r*_*u*_ is the ratings set of user *u*, *r*_*ui*_ is the rating of user *u* on item *i*, and *x*_*u*_ is mean genre ratings of user *u*.
3. *Clustering*: Line 15 to 19 calculate the Pearson Similarity using Eq (6). The initial membership is calculated in Line 20 to 26, i.e., if a user has ever watched a movie in the genre *g*_*j*_, then $m_{uj}^{\prime }=1$, otherwise it is 0. In Line 27, the FCM clustering is applied to get the membership value of each user corresponding to each cluster.
4. *Similarity calculation (Line 28−Line 34)*: The Similarity among users in clusters C is calculated which is defined in Eq (7).
5. *Group Formation (Line 35−Line 40)*: After Similarity Matrix computation, Top-k similar users are selected to create a group.

**Algorithm 1** Pseudocode for Group Formation

  **Input**: *ratings*(*R*), *items*(*I*), *k-users*;

  **Output**: Groups

1: *J* ← set of genres(*I*)

2: *X* ← 0

3: *M*′ ← 0

4: *V* ← *getInitialClusterCenter*()

5: **for each** user *u* ∈ *U* **do**

6:   **for each** item *i* ∈ *r*_*u*_ **do**

7:    **for each** genre *j* ∈ *J* **do**

8:     **if** i *contains* j **then**

9:      *x*_*uj*_+ = *r*_*ui*_

10:     **end if**

11:    **end for**

12:    $x_{u}=\frac{x_{u}}{\left|r_{u}\right|}$

13:   **end for**

14: **end for**

15: **for each** user *u* ∈ *U* **do**

16:   **for each** user *v* ∈ *U* **do**

17:    *Sim*_(*u*,*v*)_ ← *PCC*_(*u*,*v*)_

18:   **end for**

19: **end for**

20: **for each** user *u* ∈ *U* **do**

21:   **for each** genre *j* ∈ *J* **do**

22:    **if** *x*_*uj*_ ≠ 0 **then**

23:     $m_{uj}^{\prime }=1$

24:    **end if**

25:   **end for**

26: **end for**

27: *M* ← *FCM*(*V*, *M*′, *X*)

28: **for each** user *u* ∈ *U* **do**

29:   **for each** user *v* ∈ *U* **do**

30:    **for each** cluster *c* ∈ *C* **do**

31:     *S*_(*u*,*v*,*c*)_ = *Sim*_(*u*,*v*)_ × *M*_(*v*,*c*)_

32:    **end for**

33:   **end for**

34: **end for**

35: **for each** user *u* ∈ *U* **do**

36:   **for each** cluster *c* ∈ *C* **do**

37:    *G* ← *getSorted*(*S*, *k-users*)

38:   **end for**

39: **end for**

40: **return** *G*

In the following, we present an illustrative example of the proposed system.

### Illustrative example

A sample dataset consisting of 10 users and 7 movies is shown in Table 3. The information about movie genres is presented in Table 4. Users’ similarity computed with Eq (6) is shown in Table 5, whereas Table 6 indicates user to cluster score using FCM. The Similarity computation between users in cluster 1 and cluster 2 is presented in Tables 7 and 8, respectively. The similarity between user *u* and *v* in a cluster *c* is computed using Eq (7), i.e., by taking the product of Pearson Similarity between *u* and *v*, and membership of *v* in cluster *c*. Assuming a user *u*_3_ and cluster *c*_1_, the actual ratings of Top-k similar users to *u*_3_ are presented in Table 9. After forming the group, the NCF is applied to predict the ratings of unrated items, as shown in Table 10. Average aggregation strategy is applied on predicted ratings to generate Top-k recommendations, as shown in Table 11.

**Table 3:** Rating matrix.

	*i*_1_	*i*_2_	*i*_3_	*i*_4_	*i*_5_	*i*_6_	*i*_7_
***u*_1_**	5	4	−	5	3	4	−
***u*_2_**	−	4	5	4	−	5	2
***u*_3_**	4	3	3	5	3	5	−
***u*_4_**	4	5	−	3	3	−	−
***u*_5_**	5	3	−	5	3	4	2
***u*_6_**	4	2	2	−	2	5	3
***u*_7_**	−	5	3	4	4	−	3
***u*_8_**	3	−	−	4	3	3	−
***u*_9_**	4	5	4	−	5	4	−
***u*_10_**	4	5	−	4	3	−	4

**Table 4:** Genres.

	*Action*	*Comedy*	*Thriller*	*Fiction*
*i*_1_	1	0	1	0
*i*_2_	0	1	1	0
*i*_3_	0	0	1	1
*i*_4_	1	1	1	0
*i*_5_	0	1	0	1
*i*_6_	1	1	0	0
*i*_7_	1	0	0	1

**Table 5:** Pearson similarity.

	*u*_1_	*u*_2_	*u*_3_	*u*_4_	*u*_5_	*u*_6_	*u*_7_	*u*_8_	*u*_9_	*u*_10_
*u*_1_	0.00	−0.14	0.76	0.69	0.91	0.00	−0.19	0.73	0.17	0.34
*u*_2_	−0.14	0.00	0.28	−0.38	−0.29	−0.11	0.10	−0.26	−0.09	−0.49
*u*_3_	0.76	0.28	0.00	0.26	0.59	−0.00	−0.31	0.73	0.25	−0.27
*u*_4_	0.69	−0.38	0.26	0.00	0.52	−0.31	0.33	0.22	0.31	0.74
*u*_5_	0.91	−0.29	0.59	0.52	0.00	0.08	−0.31	0.80	−0.13	0.42
*u*_6_	0.00	−0.11	−0.00	−0.31	0.08	0.00	−0.82	−0.02	0.34	−0.37
*u*_7_	−0.19	0.10	−0.31	0.33	−0.31	−0.82	0.00	−0.34	−0.10	0.44
*u*_8_	0.73	−0.26	0.73	0.22	0.80	−0.02	−0.34	0.00	−0.07	−0.00
*u*_9_	0.17	−0.09	0.25	0.31	−0.13	0.34	−0.10	−0.07	0.00	−0.25
*u*_10_	0.34	−0.49	−0.27	0.74	0.42	−0.37	0.44	−0.00	−0.25	0.00

**Table 6:** FCM.

	*c*_1_	*c*_2_
*u*_1_	0.612458	0.387542
*u*_2_	0.422801	0.577199
*u*_3_	0.575912	0.424088
*u*_4_	0.556497	0.443503
*u*_5_	0.724817	0.275183
*u*_6_	0.516828	0.483172
*u*_7_	0.392983	0.607017
*u*_6_	0.471901	0.528099
*u*_9_	0.418408	0.581592
*u*_10_	0.425203	0.574797

**Table 7:** Similarity cluster 1.

	*u*_1_	*u*_2_	*u*_3_	*u*_4_	*u*_5_	*u*_6_	*u*_7_	*u*_8_	*u*_9_	*u*_10_
*u*_1_	−	−0.05	0.44	0.38	0.66	0.00	−0.07	0.34	0.07	0.14
*u*_2_	−0.08	−	0.16	−0.21	−0.21	−0.05	0.04	−0.12	−0.04	−0.20
*u*_3_	0.47	0.11	−	0.14	0.42	−0.00	−0.12	0.34	0.10	−0.11
*u*_4_	0.42	−0.16	0.15	−	0.38	−0.16	0.12	0.10	0.13	0.31
*u*_5_	0.55	−0.12	0.34	0.29	−	0.04	−0.12	0.37	−0.05	0.17
*u*_6_	0.00	−0.04	−0.00	−0.17	0.06	−	−0.32	−0.01	0.14	−0.15
*u*_7_	−0.11	0.04	−0.18	0.18	−0.23	−0.42	−	−0.16	−0.04	0.18
*u*_8_	0.45	−0.11	0.42	0.12	0.58	−0.01	−0.13	−	−0.03	−0.00
*u*_9_	0.10	−0.04	0.14	0.17	−0.09	0.18	−0.04	−0.03	−	−0.10
*u*_10_	0.20	−0.20	−0.15	0.41	0.30	−0.19	0.17	−0.00	−0.10	−

**Table 8:** Similarity cluster 2.

	*u*_1_	*u*_2_	*u*_3_	*u*_4_	*u*_5_	*u*_6_	*u*_7_	*u*_8_	*u*_9_	*u*_10_
*u*_1_	−	−0.08	0.32	0.30	0.25	0.00	−0.11	0.39	0.10	0.19
*u*_2_	−0.05	−	0.11	−0.17	−0.08	−0.05	0.06	−0.13	−0.05	−0.28
*u*_3_	0.29	0.16	−	0.11	0.16	−0.00	−0.19	0.38	0.14	−0.15
*u*_4_	0.26	−0.22	0.11	−	0.14	−0.15	0.20	0.12	0.18	0.42
*u*_5_	0.35	−0.16	0.25	0.23	−	0.04	−0.19	0.42	−0.07	0.24
*u*_6_	0.00	−0.06	−0.00	−0.14	0.02	−	−0.50	−0.01	0.20	−0.21
*u*_7_	−0.07	0.06	−0.13	0.14	−0.08	−0.39	−	−0.18	−0.06	0.25
*u*_8_	0.28	−0.15	0.31	0.10	0.22	−0.01	−0.21	−	−0.04	−0.00
*u*_9_	0.06	−0.05	0.10	0.14	−0.03	0.16	−0.06	−0.04	−	−0.14
*u*_10_	0.13	−0.28	−0.11	0.32	0.11	−0.18	0.26	−0.00	−0.14	−

**Table 9:** Actual group members ratings.

	*i*_1_	*i*_2_	*i*_3_	*i*_4_	*i*_5_	*i*_6_	*i*_7_
*u*_1_	5	4	−	5	3	4	−
*u*_3_	4	−	3	4	3	2	−
*u*_4_	4	5	−	3	3	−	−
*u*_5_	2	3	−	−	3	4	2
*u*_8_	3	−	−	4	3	3	−

**Table 10:** Predicted group members ratings.

	*i*_1_	*i*_2_	*i*_3_	*i*_4_	*i*_5_	*i*_6_	*i*_7_
*u*_1_	5	4	**3**	5	3	4	**3**
*u*_3_	4	**5**	3	4	3	2	**3**
*u*_4_	4	5	**3**	3	3	**4**	**4**
*u*_5_	2	3	**2**	**5**	3	4	2
*u*_8_	3	**4**	**2**	4	3	3	**2**

**Table 11:** Predicted group ratings.

	*i*_1_	*i*_2_	*i*_3_	*i*_4_	*i*_5_	*i*_6_	*i*_7_
**group ratings**	3.6	4.2	2.6	4.2	3.0	3.4	2.8

## Performance evaluation

In this section, we present the performance evaluation of proposed HTGF. The MovieLens 1M dataset [30] is used to evaluate the effectiveness of proposed model. The dataset includes 6040 users, 3900 movies, and 1,000,209 ratings. Every user rated at least 20 movies in MovieLens dataset. The movies include 19 different genres. MovieLens 1M is a standard dataset widely used by researchers in movie recommender systems as it contains rich feature set of movies, and users’ historical ratings information that are required to properly train a model to perform movie recommendations. MovieLens 1M is a standard dataset widely used by researchers in recommendation systems. We compare our work with baseline models i.e., ALS and SVD, which are used by most of the existing schemes for comparisons in group recommendation scenarios [25]

- The ALS algorithm factorizes a given matrix *R* into two factors *U* and *V*, such that *R* ≈ *U*^*T*^*V*. Here, *U* represents set of users and *V* represents set of movies. The unknown row dimension is given as a parameter to the algorithm and is called latent factors. The *i*th column of the user matrix is denoted by *u*_*i*_ and the *i*th column of the movie matrix is *v*_*i*_. The matrix *R* can be called the ratings matrix with (*R*)_*i*,*j*_ = *r*_*i*,*j*_. Further details on ALS can be found in [66].
- The SVD matrix factorization method maps users and movies to a joint latent factor space of dimensionality *f*. A user *u* is associated to a row vector represented by *p*_*u*_ ∈ *R*^*f*^, and a movie *v* is associated with a column vector given by *q*_*u*_ ∈ *R*^*f*^. A user’s estimated rank for a movie *v* is represented as ${\hat{r}}_{u,v}=q_{v}^{T}\times p_{u}$. More details about SVD can be found in [67].

### Performance metrics

A user’s rating for a movie ranges from [1, 5], where 1 being lowest and 5 being highest. To evaluate the performance of HTGF, we considered RMSE, MAE, Precision, and Recall as traditional performance comparison benchmarks [68]. According to [69], we can evaluate a recommender system in two measures: (a) prediction accuracy and (b) classification accuracy. Prediction accuracy means how correctly our model predicts the ratings. For this, we use RMSE and MAE. Whereas classification accuracy quantifies the correctness of recommendations, and this includes Precision and Recall. We also calculate satisfaction in order to evaluate the effectiveness of recommendations. Following are the performance measures.

RMSE: It is a criterion for calculating the error. It can be defined as follows.

$$\begin{array}{c} RMSE=\sqrt{\frac{\sum_{i=1}^{n}{\left({\hat{y}}_{ui}-y_{ui}\right)}^{2}}{N}}. \end{array}$$ (11)

MAE: It is the absolute difference between predicted rating and actual rating. It can be represented as follows.

$$\begin{array}{c} MAE=\frac{\sum_{i=1}^{n}|{\hat{y}}_{ui}-y_{ui}|}{N}. \end{array}$$ (12)

Precision: Precision is used to evaluate the recommended movies that are relevant to users. It is defined as the fraction of *hits*_*u*_ It can be defined as follows.

$$\begin{array}{c} Precision_{u}=\frac{\left|hits_{u}\right|}{\left|recset_{u}\right|}. \end{array}$$ (13)

Where *hits*_*u*_ is the number of correctly recommended movies that are relevant to user *u*, and *recset*_*u*_ is the set of Top-k recommended movies.

Recall: Recall is used to evaluate the fraction of instances over the total number of relevant recommendations. It can be defined as follows.

$$\begin{array}{c} Recall_{u}=\frac{\left|hits_{u}\right|}{\left|rel_{u}\right|}. \end{array}$$ (14)

F1-Score: F1-score is used to evaluate the quality of HTGF. It can be calculated as follows.

$$\begin{array}{c} \text{F1-Score}=\frac{2\times Precision_{u}\times Recall_{u}}{Precision_{u}+Recall_{u}}. \end{array}$$ (15)

Group Satisfaction: Group satisfaction measure is used to evaluate the group satisfaction for the recommended Top-k movies. Group satisfaction is denoted as follows:

$$\begin{array}{c} Sat(g,R)=\frac{\sum_{u\in g}Sat(u,R)}{\left|g\right|}. \end{array}$$ (16)

Where *g* is the group, and *R* is the set of recommended movies. It is calculated by the average individual user satisfaction. |*g*| is the total number of members in the group [70].

$$\begin{array}{c} Sat(u,R)=\frac{\sum_{i\in R}{\hat{y}}_{ui}}{\left|R\right|}. \end{array}$$ (17)

The objective function is to maximize the group satisfaction. For this, we have to maximize the individual satisfaction on recommended movies.

### Parameters setting

Table 12 summarizes the values of different parameters used in the proposed model. To prevent model from overfitting early stopping is used which sets epoch size to 20, and batch size to 64. A combination of Adam optimizer, Sigmoid, and Binary Cross Entropy was used which penalize the wrong predicted ratings. Group size ranges from 5 to 30, and Top-5 movies were recommended to groups.

**Table 12:** Values of parameters in the proposed model.

Parameter	Value
Epoch size	20
Batch size	64
Learning rate	Adjusted to 0.001
Optimizer	Adam
Activation Function	Sigmoid
Loss Function	Binary Cross Entropy
Group size	[5, 10, 15, 20, 25, 30]
Top-k Recommendations	5

### Results

The efficiency of the proposed group recommender model is measured through group satisfaction metric. We split our dataset into 80 − 20, 80% ratings are used to train the model, and 20% ratings are used to evaluate the model. Performance of the proposed model was assessed by using RMSE, MAE, Recall, Precision, F1-Score, and group satisfaction measure. RMSE and MAE indicate the prediction accuracy of the model, while precision and recall are used to evaluate the group recommendations generated by the model. We calculate the effect of group satisfaction by varying the group size. Table 13 provides the comparison with existing models.

**Table 13:** Results.

Models	RMSE	MAE	Precision	Recall	F1-Score
HTGF	0.7759	0.6021	1.0	0.0653	0.1226
SVD	0.8244	0.6534	0.9440	0.0616	0.1156
ALS	0.8761	0.6633	0.8960	0.0603	0.1130

We used average aggregation strategy to aggregate the group members’ preferences. It is consensus-based strategy, and considers the preferences of all group members, unlike Most Pleasure strategy, which is a veto-based strategy. Lower values of RMSE and MAE indicate that the predicted ratings are close to the actual ratings. Higher the precision and recall means more relevant the recommendations are. We have also calculated the preferences of user for cluster 2, which is described in Table 14. It is observed that same user has different preferences in different groups.

**Table 14:** Cluster 2 results.

Models	RMSE	MAE	Precision	Recall	F1-Score
HTGF	0.7808	0.6096	0.4200	0.0182	0.0349

**Fig 4:**
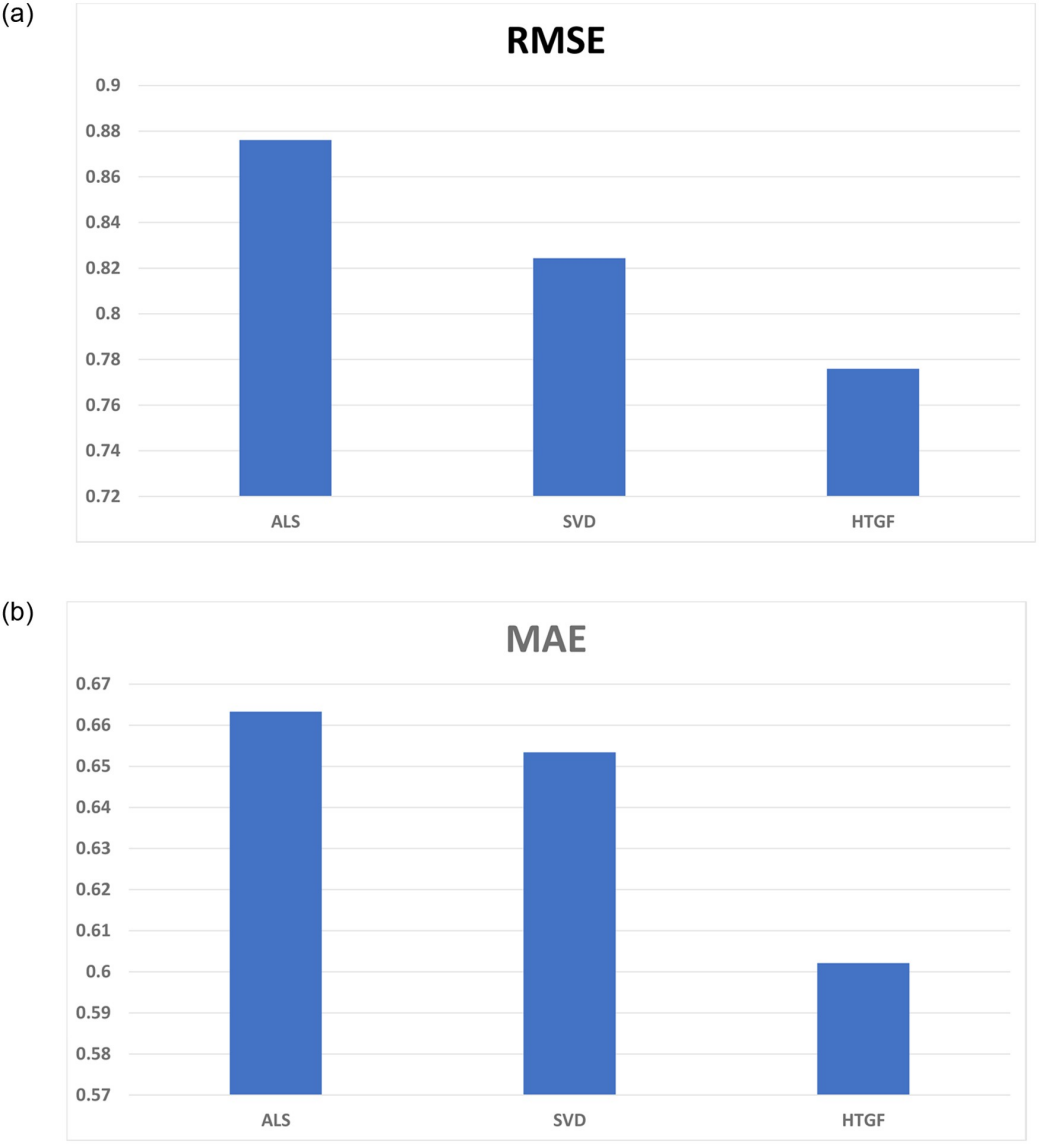
Prediction accuracy. (a) root mean square error and (b) mean absolute error.

In Fig 4(a), the proposed model is compared with the existing approaches based on RMSE. The RMSE of ALS and SVD is 0.8761 and 0.8244, respectively, and of HTGF is 0.7759. Lower RMSE means the model’s predicted ratings are close to the actual ratings. Whereas, in Fig 4(b) the comparison is based on MAE, which is for ALS and SVD is 0.6633 and 0.6534, respectively, and the MAE of HTGF is 0.6021. Lower MAE means higher the accuracy of model.

**Fig 5:**
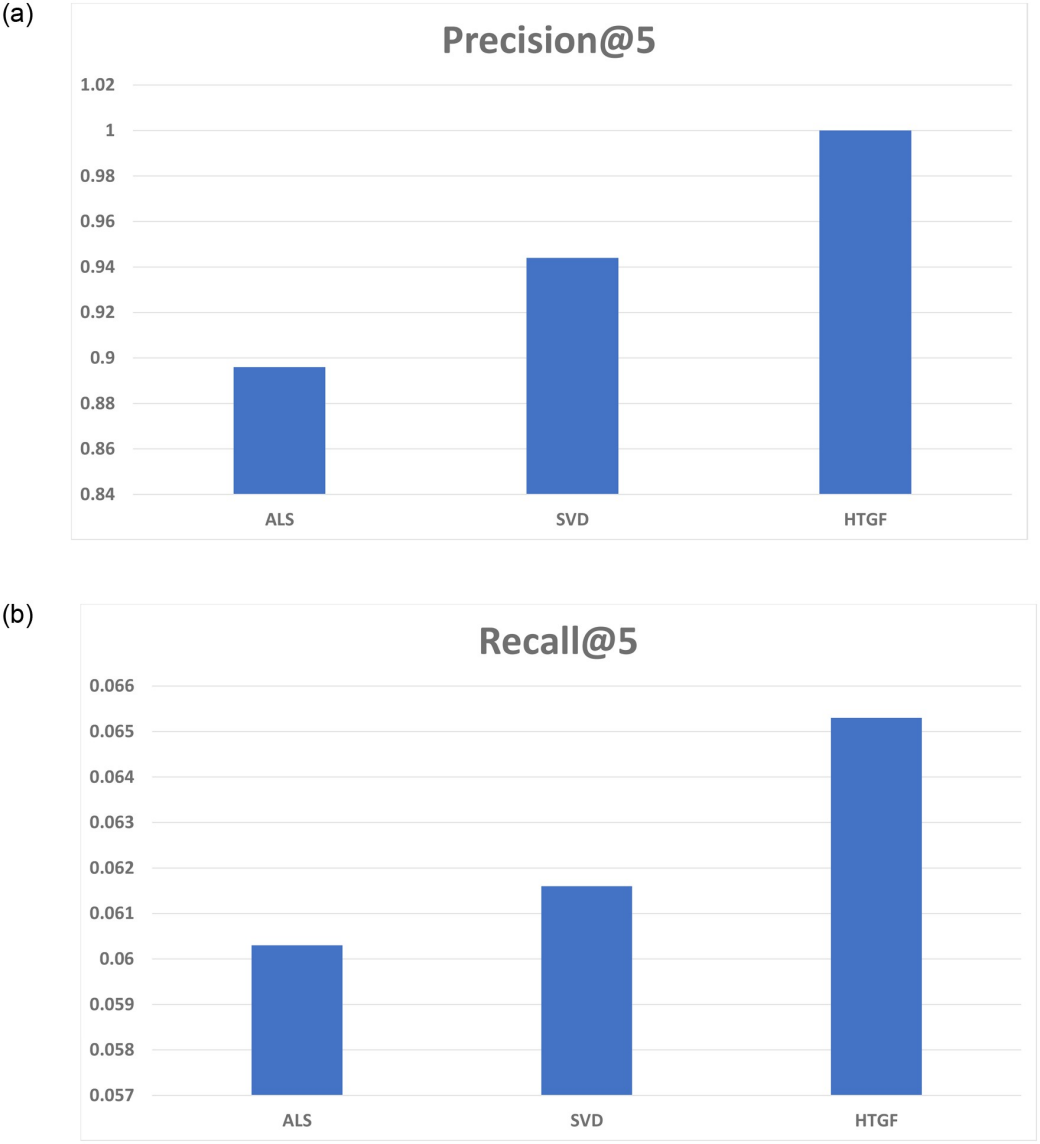
Classification accuracy. (a) precision and (b) recall.

In Fig 5(a), the comparison is based on Precision, which is 0.8960, 0.9440, and 1.0 for ALS, SVD, and HTGF, respectively. The comparison based on recall is described in Fig 5(b). The values of recall for ALS, SVD, and HTGF are 0.0603, 0.0616, and 0.0653, respectively. Higher value of recall means greater coverage and more relevant the recommendations are. ALS and SVD indicate low performance in terms of precision and recall as their estimation mechanisms are sensitive to data sparseness. On the contrary, HTGF is trained on NCF Framework which utilizes the latent factors of users and items due to which it is not significantly effected by data sparseness.

**Fig 6:**
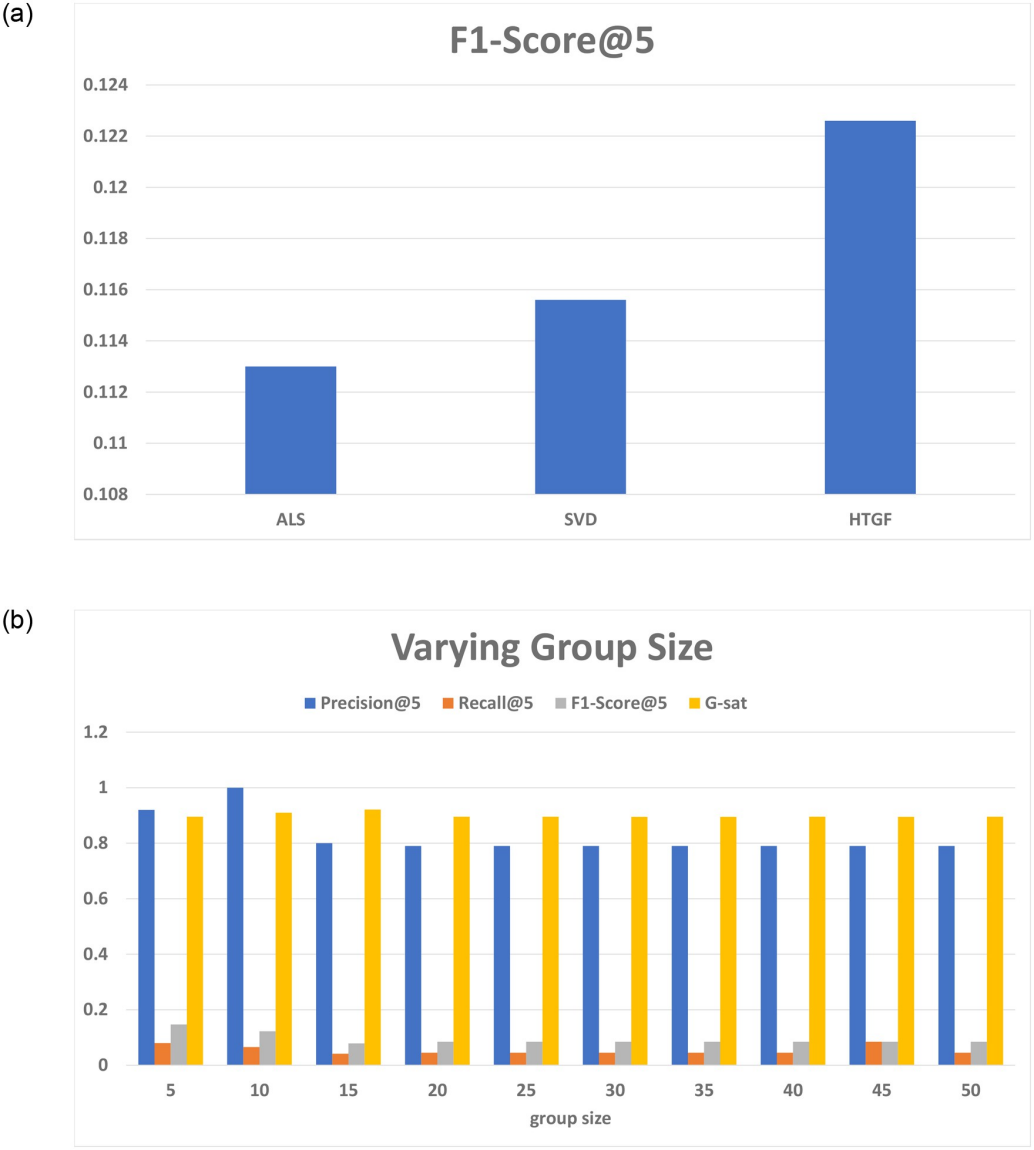
Group size. (a) F1-Score and (b) Varying Group Size.

Fig 6(a) shows the comparison based on F1-score which is 0.1130, 1156, and 0.1226 for ALS, SVD, and HTGF. The precision of HTGF is highest for k = 10. However, increase in number of group members reduces the precision and recall. Fig 6(b) shows the impact of changing group size on the proposed framework in terms of precision, recall, and group satisfaction. We observe optimum value when group size is 10, which is same as of Nawi *et al*. [25] where it is calculated through elbow method. For the group size greater than 15, the HTGF shows almost constant results better than existing schemes which experience performance degradation above group size 10, and hence their graphs are not included in the figure. The values of recall are comparatively lower than the other parameters. This is mainly because of Eq (14), in which the numerator representing the hits has lesser values. The number of hits of individual group members are comparatively smaller because of not lying in Top-k movies for a group, which lowers the overall value of recall. However, as can be observed in Table 13, the recall of HTGF is better than existing schemes.

It can be observed from the results that proposed model predicts more accurate ratings compared to existing schemes. The matrix splitting and estimation procedure of ALS results in low accuracy as compared to SVD that splits the matrices into three sub-matrices. Moreover, the ALS and SVD experience degradation in recommendation quality as they fail to capture the implicit preferences of individuals participating in a group [25]. However, our proposed model based on NCF takes into account the latent feature vectors of users and movies which minimizes the error rate as compared to ALS and SVD.

Tables 15–17 show the values for precision, recall, and F1-Score for the proposed scheme and the baselines by varying the group size. It can be observed that SVD shows better precision value for group size of 10 and greater. However, this is at the cost of lower values of recall. If we inspect the F1-Score of the three schemes, we can observe that the F1-Score of the proposed HTGF scheme is better than ALS and SVD. This is because, F1-Score formula incorporates the values of both precision and recall. It is noteworthy that some models gives high precision but they give low recall value so in order to find the more accurate results F1-score is used.

**Table 15:** Performance of HTGF by varying group size.

Group Size	5	10	15	20	25	30
**Precision@5**	0.9199	1.0	0.7999	0.7900	0.7900	0.7900
**Recall@5**	0.0798	0.0653	0.0415	0.0448	0.0448	0.0448
**F1-Score**	0.1468	0.1226	0.0789	0.0848	0.0848	0.0848

**Table 16:** Performance of ALS by varying group size.

Group Size	5	10	15	20	25	30
**Precision@5**	0.9199	0.8960	0.92	0.9300	0.9300	0.9400
**Recall@5**	0.0146	0.0603	0.0207	0.0175	0.0175	0.0175
**F1-Score**	0.0288	0.1130	0.0406	0.0344	0.0344	0.0344

**Table 17:** Performance of SVD by varying group size.

Group Size	5	10	15	20	25	30
**Precision@5**	0.9199	0.9440	0.92	0.9400	0.9400	0.9400
**Recall@5**	0.0146	0.0616	0.0123	0.0133	0.0133	0.0133
**F1-Score**	0.0288	0.1156	0.0243	0.0264	0.0264	0.0264

### Statistical analysis

In this subsection, we present the statistical significance of the results obtained previously. To find the statistical significance, we follow an approach similar to the one presented in [71]. We have checked the resulting values based on parameters, such as precision, recall, and F1-score and found their distribution is normal. In that case, there is a need for a parametric test that involves two variables so that we can compare our proposed scheme with the baselines. Amongst the various available options, we selected the most popular *t*-test to compute the significance level *p* with threshold value set as *p* < 0.05. We define the following hypothesis:

H0: HTGF and baseline model have no difference.
H1: A significant difference exists between HTGF and baseline models.

Table 18 presents the mean, standard deviation (SD), and *p*-value for the performance values obtained in Tables 15 and 17. It can be observed that the *p*-value for the parameter F1-Score is less than the significance level threshold, i.e., *p* < 0.05, which means that the significant difference exists among the values of HTGF and other baselines. So, we can reject the null hypothesis and accept the alternate hypothesis.

**Table 18:** Statistical comparison of HTGF with baselines.

	HTGF	SVD	ALS
**Precision**			
**Mean**	0.8483	0.9339	0.9226
**SD**	0.0901	0.0109	0.0150
**p-value**	−	0.068	0.1394
**Recall**			
**Mean**	0.0535	0.0214	0.0246
**SD**	0.0155	0.0197	0.0175
**p-value**	−	0.0101	0.0161
**F1-Score**			
**Mean**	0.10045	0.0413	0.0476
**SD**	0.0277	0.0364	0.0322
**p-value**	−	0.0092	0.0149

### Error analysis

**Fig 7:**
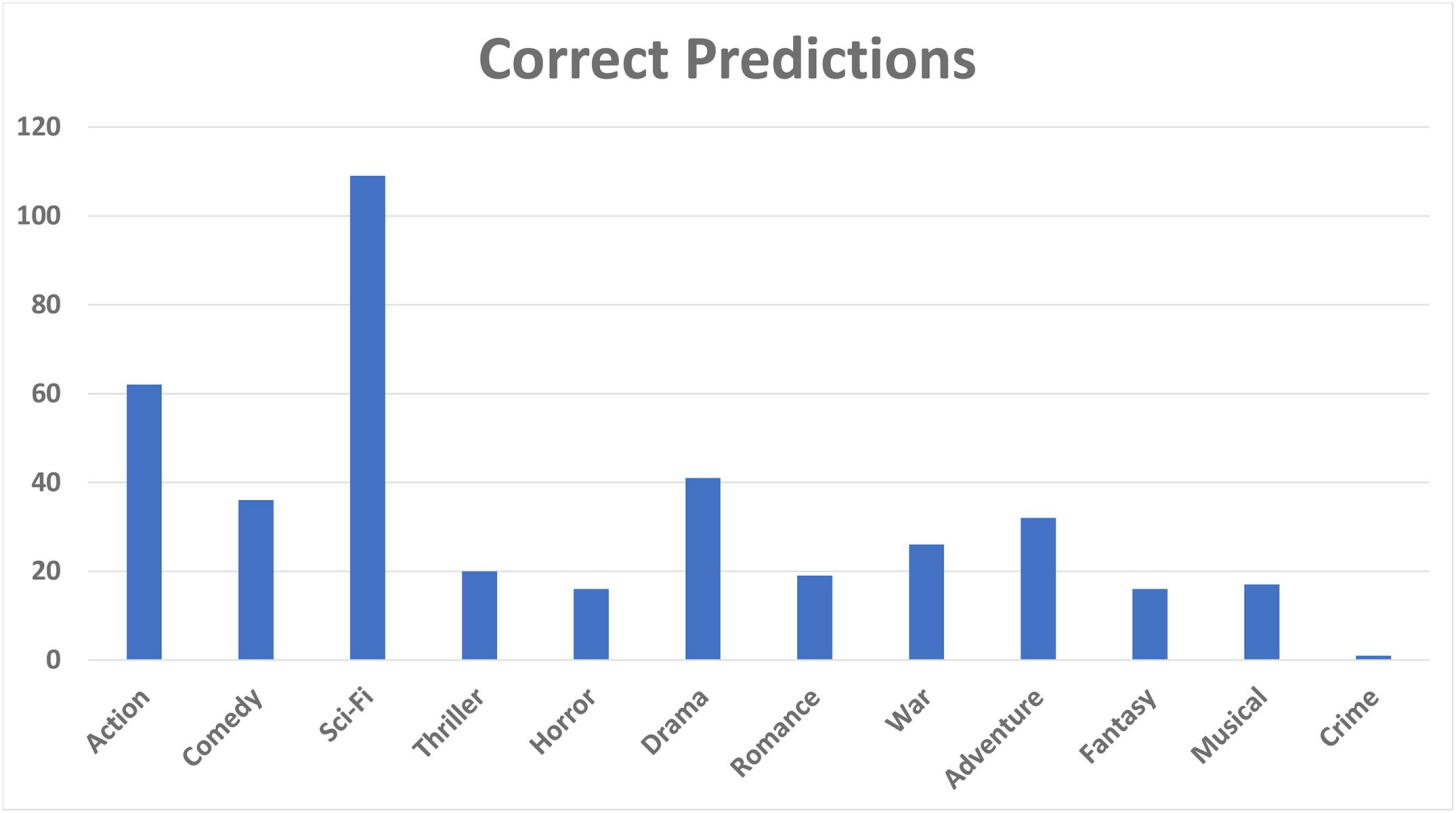
Correct predictions by HTGF.

In this subsection, we present the error analysis of our proposed model. To perform the error analysis, we follow the similar procedure discussed in [72]. We have conducted an analysis of group ratings which are predicted wrongly by our proposed model. For this purpose, we compared the proposed model’s predicted ratings with actual ratings and analyzed the data manually for finding the possible cues. As first step, we generated two csv files containing actual group ratings and predicted group ratings, and analyzed them manually. The error analysis code and csv files are uploaded on github [73]. During the analysis, we found that movies having the following genres occurring together (action, sci-fi, thriller) are usually predicted wrongly, and the movies having any of the genres (action, comedy, drama, sci-fi) are predicted correctly by our proposed model. Fig 7 shows the genre counts that are correctly predicted by our model. Most of the time, movies having genres: action, drama, comedy, fantasy, and sci-fi are popular among all group members.

**Fig 8:**
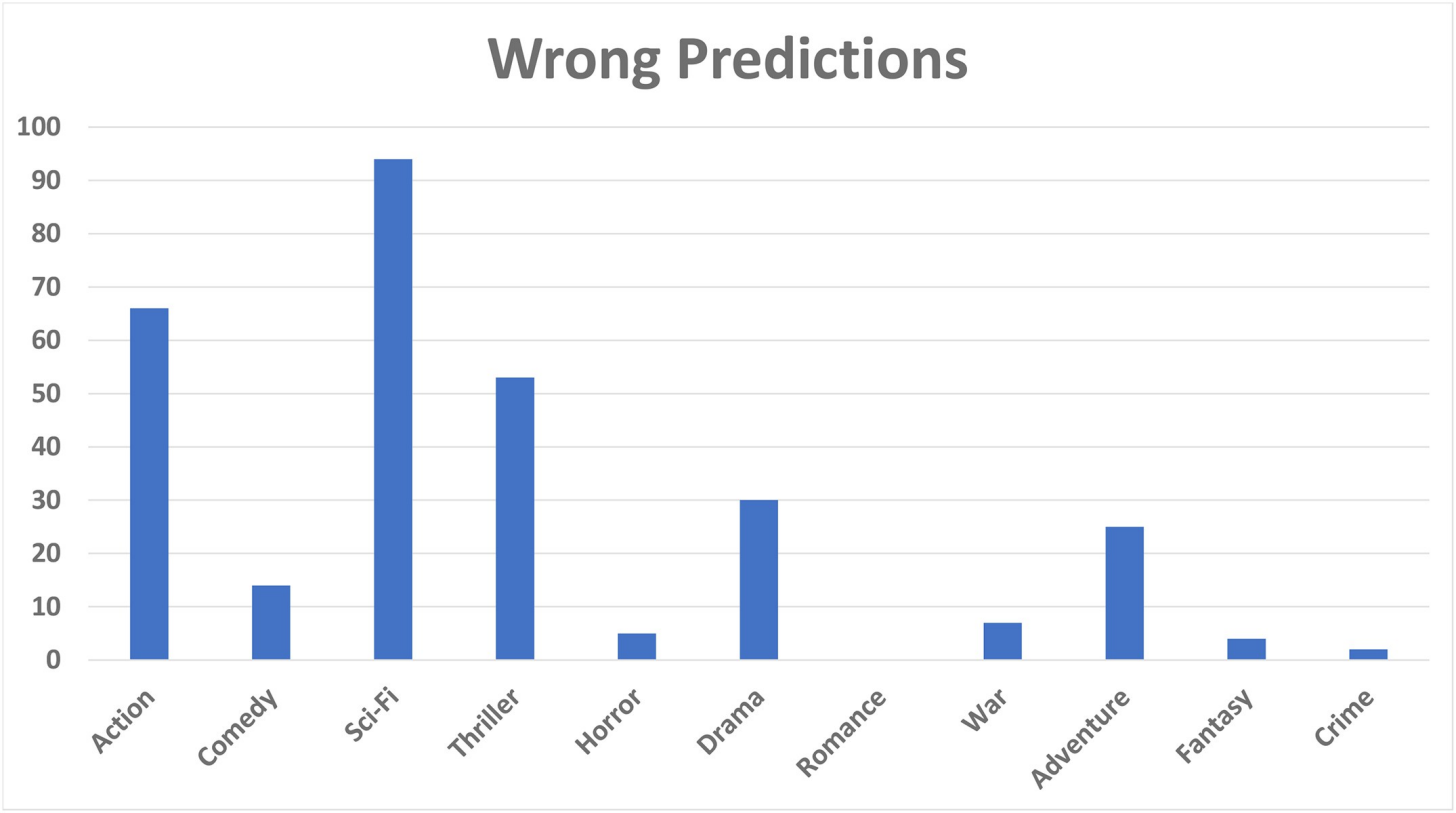
Wrong predictions by HTGF.

**Fig 9:**
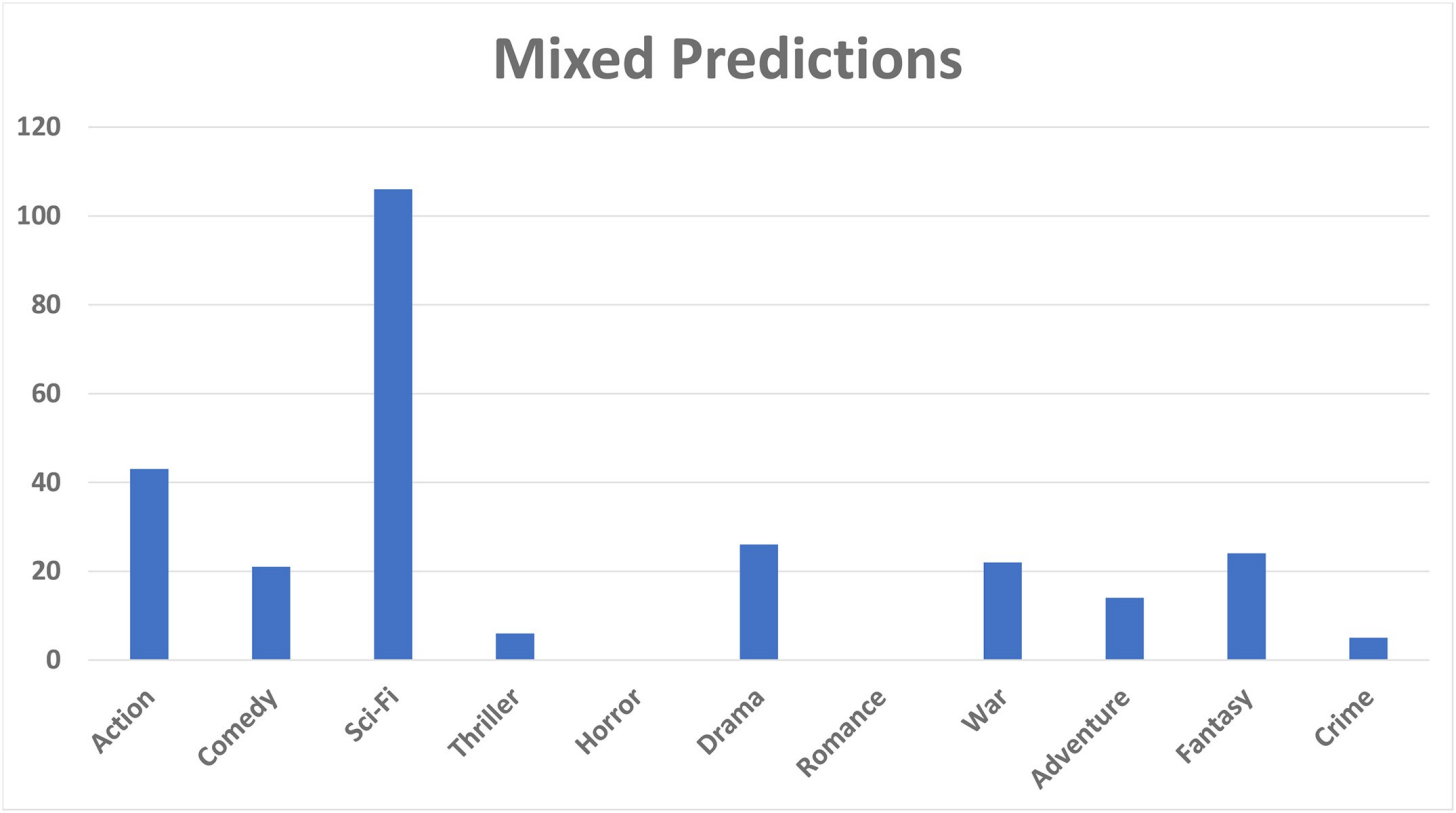
Mixed predictions by HTGF.

A pattern is found that the rating of movie having both genres (comedy, drama), or (drama, sci-fi), or (action, sci-fi) are predicted correctly by our model. Fig 8 shows the ratings that are wrongly predicted by our model. We observed that when the genres such as, action and sci-fi, occur along with thriller, our model predicts the wrong ratings. Fig 9 presents the mix ratings of our model. During manual analysis we found that our model is confused when movie has the combination of genres: (action, adventure, sci-fi) or (sci-fi, war), or (action, sci-fi, thriller). From the aforementioned discussion, we concluded that our model is not able to predict rantings when movie has genres (action, sci-fi, thriller) occurring simultaneously. Moreover, our model predicts average ratings when the movie has genre combination of (action, adventure, sci-fi), and our model predicts correct ratings when the movie has any of the following genres: (action, comedy, drama, sci-fi). In case of the genre ‘thriller’, most of the time our model predicts wrong ratings.

## Conclusion

A hybrid Two-Phase Group Recommender Framework (HTGF) is presented, and results are compared with the existing models. The proposed work integrates clustering techniques such as PCC and FCM that allows a user’s membership to different groups based on preference similarity. NCF is used to predict the ratings of unrated items of group members. NCF exhibited better performance over the counterparts as it uses the latent factors of users and items. Different from the previous work the proposed work discusses a new perspective of group formation by allowing a user to have multiple groups. It has been observed that the same user has different preferences in different groups. For instance, a user may have different preferences while watching movie with friends, than with family. The evaluation of proposed model with MovieLens-1M dataset indicates improved performance of HTGF compared to existing schemes.

In future, we intend to use multi attention neural networks instead of average aggregation strategy to recommend Top-k movies to the group. Group members can influence each other, so we will consider the influence of group members during group formation and explore its impact on final recommendations. Moreover, we are interested to explore the transformer-based methods using NLP approaches for group recommendations. Furthermore, we will test our model on multiple datasets to see the impact on performance.
